# Frontal midline theta oscillations during mental arithmetic: effects of stress

**DOI:** 10.3389/fnbeh.2015.00096

**Published:** 2015-04-20

**Authors:** Matti Gärtner, Simone Grimm, Malek Bajbouj

**Affiliations:** ^1^Affective Neuroscience and Emotion Modulation, Department of Education and Psychology, Freie Universität BerlinBerlin, Germany; ^2^Department of Psychiatry, Charité, Campus Benjamin FranklinBerlin, Germany; ^3^Department of Psychiatry, Psychotherapy and Psychosomatics, University of ZurichZurich, Switzerland

**Keywords:** acute stress, mental arithmetic, frontal theta oscillations, EEG

## Abstract

Complex cognitive tasks such as mental arithmetic heavily rely on intact, well-coordinated prefrontal cortex (PFC) function. Converging evidence suggests that frontal midline theta (FMT) oscillations play an important role during the execution of such PFC-dependent tasks. Additionally, it is well-established that acute stress impairs PFC function, and recent evidence suggests that FMT is decreased under stress. In this EEG study, we investigated FMT oscillations during a mental arithmetic task that was carried out in a stressful and a neutral control condition. Our results show late-onset, sustained FMT increases during mental arithmetic. In the neutral condition FMT started to increase earlier than in the stress condition. Direct comparison of the conditions quantified this difference by showing stronger FMT increases in the neutral condition in an early time window. Between-subject correlation analysis showed that attenuated FMT under stress was related to slowed reaction times. Our results suggest that FMT is associated with stimulus independent mental processes during the natural and complex PFC-dependent task of mental arithmetic, and is a possible marker for intact PFC function that is disrupted under stress.

## Introduction

The execution of complex cognitive tasks strongly depends on prefrontal cortex (PFC) function, and its network connections to other brain regions (Miller and Cohen, 2001). PFC function during high-level cognition include the maintenance and manipulation of information in the absence of external stimulation, the protection of these fragile representations from external and internal distractions, and the top-down control of other brain regions (Goldman-Rakic, 1995; Arnsten, 2009). Converging evidence suggests a close relationship between PFC-dependent cognitive tasks and frontal midline theta (FMT, 4–8 Hz) oscillations (for reviews see Mitchell et al., 2008; Cavanagh and Frank, 2014; Hsieh and Ranganath, 2014). High-level cognitive processes that are coincided by FMT oscillations include working memory (Jensen and Tesche, 2002; Onton et al., 2005; Itthipuripat et al., 2013; Zakrzewska and Brzezicka, 2014), episodic encoding (Klimesch et al., 1997; Nyhus and Curran, 2010; White et al., 2013) and retrieval (Klimesch et al., 2001; Osipova et al., 2006), mental arithmetic (De Smedt et al., 2009; Ishii et al., 2010, 2014), error processing (Luu et al., 2004; Cohen, 2011) and action monitoring (Cavanagh et al., 2012).

The broad range of cognitive phenomena that FMT oscillations have been related to make it challenging to interpret their functional role. Furthermore, it is questionable whether FMT fulfills the same function in all of the observed phenomena. Indeed, functional interpretations tend to focus on subsets of the described phenomena, such as cognitive control processes (Cavanagh and Frank, 2014), or processes related to memory function (Hsieh and Ranganath, 2014). One reason for different functional roles of FMT during cognitive control and memory processes is that the nature of these oscillations differs in various aspects. During cognitive control processes, it has been suggested that FMT oscillations might provide a mechanism by which the need for cognitive control is first realized, and then communicated to other brain regions (Cavanagh and Frank, 2014). This type of FMT is usually phase-locked to the presented stimulus (evoked oscillations), and of transient nature. It has been observed in a broad range of cognitive tasks that require the allocation of attentional resources, and close relationship between event-related potentials (ERP), such as the error-related negativity (ERN) and evoked FMT oscillations has been reported (Trujillo and Allen, 2007; van Driel et al., 2012). During working memory (WM), it has been suggested that FMT might be particularly relevant to maintain and manipulate information in the absence of external stimulation (Hsieh et al., 2011; Roux and Uhlhaas, 2014). Reports of a negative relationship between FMT and default mode network (DMN; Raichle et al., 2001) activation, have put forward the assumption that FMT might also be relevant to focus attention on task demands, and inhibit task irrelevant information (Scheeringa et al., 2009). FMT during WM has been found to increase with memory demands (Gevins et al., 1997; Jensen and Tesche, 2002), is not phase-locked to presented stimuli (induced oscillations), and usually of sustained nature. A few studies directly compared evoked and the induced FMT responses and found that only the induced response is modulated by increased WM demands (Missonnier et al., 2006; Deiber et al., 2007). In these studies, the evoked response was equally present in all task conditions that required focused attention, and was absent only in a passive viewing condition.

Because of the different cognitive phenomena that FMT oscillations have been involved in, is has been suggested that FMT might provide a general mechanism the PFC uses to establish network connections to other brain regions (Anderson et al., 2010; Cohen, 2011). It is well-established that the neurochemical changes that occur during acute stress disrupt PFC network connections (Arnsten, 2009; McEwen and Morrison, 2013). Therefore, it can be assumed that FMT oscillations are affected by stress. In a recent study, first evidence has been provided that FMT oscillations during a WM task are decreased under stress (Gaertner et al., 2014). However, in the mentioned study a continuous WM paradigm (n-back task) was applied. In this task, information has to be maintained and updated continuously, which makes it difficult to clearly separate distinct mental processes in the time course of FMT oscillations.

Here, we applied a mental arithmetic task and a stress induction procedure to investigate FMT oscillations during a natural, complex cognitive task. Mental arithmetic induces workload, and naturally combines the three components (i.e., central executive, phonological loop, and visuo-spatial sketchpad) of the WM model proposed by Baddeley (Baddeley, 2003; Imbo et al., 2007). Our task consisted of moderately difficult addition equations that had to be solved one after another and were preceded by a pre stimulus baseline period. The rather long trial durations (3–10 s) allowed for a detailed time course analysis. We hypothesized FMT increases during the mental arithmetic phase compared to a pre-stimulus baseline, and decreases of performance and FMT during stress.

## Materials and methods

### Subjects and procedure

Thirty one healthy male subjects (age range: 20–50 years, mean = 31.90, *SD* = 8.31) participated in the study. They were recruited from a large database available to our research group (for details see Fuge et al., 2014). All subjects in the database were screened for psychiatric disorders, using the short version of the Structured Clinical Interview for Diagnostic and Statistical Manual of Mental Disorders (DSM-IV, SCID). Subjects showing any type of psychiatric symptoms were not included in the database. The study was carried out in accordance with the declaration of Helsinki and approved by the local ethical review board. Information regarding the violent content of the film clips was provided before the study. All subjects signed a written consent after the experiment had been explained to them.

All subjects arrived between 1 and 3 p.m. in the EEG laboratory. Upon arrival they were seated in front of a computer screen where the preparation of the EEG cap and the completion of the experimental tasks took place. During the preparation phase, subjects practiced the experimental tasks to reduce order effects. They underwent a stress- and a neutral condition that were separated by a 20 min break and counterbalanced across subjects. In each condition, subjects completed a mental arithmetic task that consisted of adding three digit numbers. The task lasted about 10 min and was preceded by a short film clip with either stressful or neutral content. Before the film clip and after the task salivary cortisol and subjective affect ratings (PANAS, Krohne et al., 1996) were assessed to control for the effectiveness of the stress induction. The mental arithmetic task was followed by two other tasks, one of which is reported in Gaertner et al. (2014).

### Task

The mental arithmetic task consisted of 50 trials in both conditions. Each trial started with the presentation of a fixation cross that was displayed for 2 s. It was followed by the presentation of a moderately difficult addition equation in which two 3-digit numbers had to be added. Equations were presented together with a result and subjects had to judge whether the presented result was correct or not. Subjects indicated their response with a mouse click on a “yes” or “no” button. After the button press the trial terminated, and if no response was received within 10 s, the experiment continued automatically with the next trial. Subjects were asked to respond as quickly and as accurately as possible, and they received no feedback whether their response was correct or not.

### Stress induction

Acute stress was induced through film clips with strongly aversive content, showing extreme violence against humans. For the neutral control condition, a set of matched neutral clips was used. The negative film clips were taken from the commercially available French movie “Irréversible” by Gaspard Noé, and the neutral scenes were taken from the movie “Comment j′ai tué mon père” by Anne Fontaine. This stress induction procedure has been shown to effectively induce stress in several published studies (e.g., Qin et al., 2009; Ossewaarde et al., 2010; Hermans et al., 2011). To involve subjects as much as possible in the film scenes and thereby increase the stress inducing effect, a self-referencing instruction was presented before the film clips. Neutral scenes were selected to be equal in luminance and similar in speech and human face presence.

### Physiological and subjective measurements

To control for the effectiveness of the stress manipulation, salivary cortisol and subjective ratings were assessed at baseline and after the task in both conditions. Saliva was sampled with salivette collection devices and samples were stored at −20°C until analysis. The analysis was carried out at the department of biopsychology in Dresden, where samples were prepared for analysis by centrifuging at 1500 × g for 5 min. Salivary-free cortisol concentrations were determined with a chemiluminescence assay with high sensitivity of 0.16 ng/mL (IBL). For details of the procedure see Dressendorfer et al. (1992). The subjective mood state was assessed by obtaining positive and negative affect ratings, using the German version of the Positive and Negative Affect Schedule (PANAS; Krohne et al., 1996). All subjective ratings were obtained during saliva sampling.

### EEG recording

EEG data was recorded from 31 Ag/AgCl scalp electrodes (BrainCap32, Easycap, Herrsching-Breitbrunn, Germany), arranged according to the extended 10–20 system. Vertical eye movements and blinks were additionally recorded from an electrode placed below the right eye. An electrode placed on the nose tip served as common reference for all channels. Signals were amplified using an analog 10 s time constant as highpass filter and then digitized with a sampling rate of 1000 Hz (BrainAmpMR plus, Brain Products, Gilching, Germany).

### EEG preprocessing

EEG data analysis was carried out in MATLAB (Version R2010b, The MathWorks, Inc., Massachsetts, USA), using custom scripts as well as the EEGLAB (Version 9.0.4, Delorme and Makeig, 2004) and the Fieldtrip (Build 20140303, Oostenveld et al., 2011) toolboxes. In the first preprocessing step the EEG data of the mental arithmetic task from both conditions was merged, re-referenced to a common average reference, resampled to 200 Hz, and 1 Hz highpass filtered. Next, an infomax ICA (Bell and Sejnowski, 1995) was applied, and eye movement related components were identified by visual inspection and removed from the data. A surface Laplacian filter was applied to reduce the effect of volume conduction (Hjorth, 1975). In the next step, the EEG data was segmented into stimulus-locked epochs (−2 to 4 s relative to stimulus onset), and response-locked epochs (−3 to 1 s relative to response). Trials were flagged as bad and discarded from further analyses if one or more of the following criteria applied: 1. A trial duration < 3 s (indicating a random response) 2. A trial duration of 10 s (no response was given) 3. Occurrence of EEG data exceeding a range of ±100 μV (artifactual data). Appliance of these criteria led to an average rejection rate of 17.2% (*SD* = 14.3%).

### Time-frequency analyses

Time-frequency representations for each subject, condition and electrode were calculated using zero-padded, hanning tapered Morlet wavelet decomposition, as implemented in EEGLAB newtimef() function. We calculated power for 99 linear-spaced frequencies ranging from 1 to 50 Hz, and 200 (167) linearly spaced time bins (advanced in 30 ms steps) across the stimulus-locked and response-locked epochs. To account for the trade-off between frequency and temporal resolution, the wavelets were modified, such that one cycle was used at the lowest frequency (1 Hz), increasing linearly to 25 cycles at the highest frequency (50 Hz). Time-frequency representations were convolved with a Gaussian kernel (1 Hz × 500 ms, FWHM), and stimulus-evoked (phase-locked) responses were removed from the data by subtracting for each condition the average waveform from the waveform of each individual trial before applying the time-frequency transform. Finally, the mean logpower of a baseline period (−1000 to 0 s relative stimulus onset) was subtracted at each frequency. To investigate possible effects of ERPs on the theta frequency range, control analyses without the removal of stimulus-evoked responses was conducted.

### Statistical analyses

Statistical analysis on the time-frequency data for all electrodes and conditions was performed using a cluster-based permutation approach (Maris and Oostenveld, 2007). Three-dimensional statistical parametric maps (*t*-test, alpha = 0.05) were calculated in the electrode–time–frequency space (for details of the procedure see Kilner et al., 2005). A cluster was defined as the sum of *t*-values in adjacent electrode–time–frequency bins. Adjacency in the electrode space was taken as a given if at least two neighboring electrodes belonged to a cluster. The alpha level for the cluster analysis was set to 0.05 (corrected) and the number of random permutations was set to 1000. For this analysis, time-frequency data was restricted to a frequency range from 1 to 8 Hz, and to time bins from 0 to 3000 ms for the stimulus-locked epochs, and −2500 to −500 ms for the response-locked epochs. Exploratory analysis was performed on a higher frequency range (8–50 Hz). In the first step, conditions were compared against baseline, and in the second step the neutral condition was compared against the stress condition.

## Results

### Behavior and peripheral physiology

**Figure 1:**
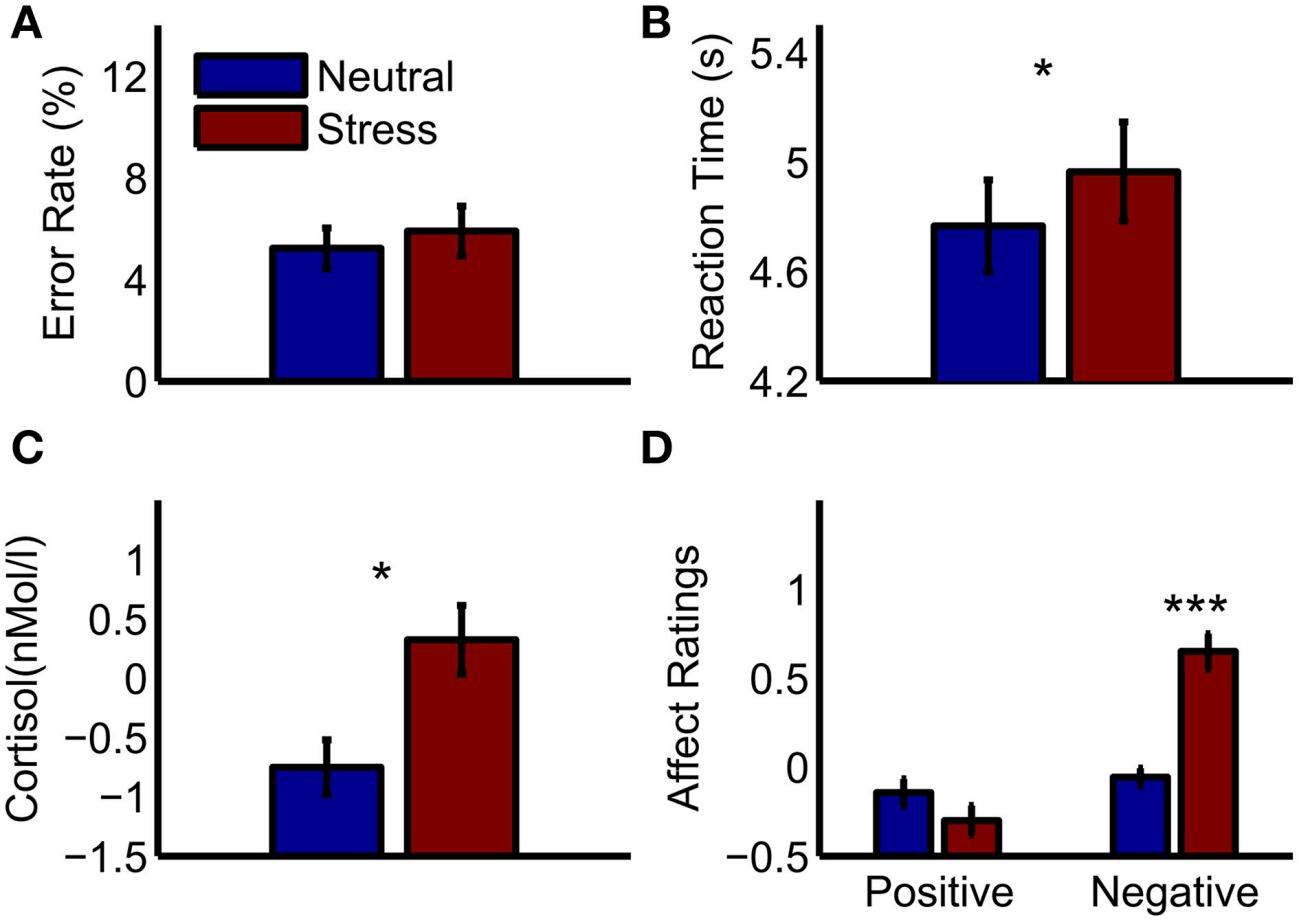
**Behavioral results and peripheral physiology. (A)** Error rates in the mental arithmetic task **(B)** reaction times in the mental arithmetic task **(C)** cortisol increases after the mental arithmetic task relative to a baseline measurement **(D)** affect ratings after the mental arithmetic task relative to baseline ratings. Significant differences between conditions are marked: ^*^p < 0.05; ^***^p < 0.001.

On average, subjects had an error rate of 11.1% during the experiment (*SD* = 9.3%), and thus performed significantly better than chance level [50%, *T*_(30)_ = −23.26, *p* < 0.001]. The average reaction time was 4.87 s (*SD* = 0.94 s). Reaction times under stress were slower than in the neutral condition [*T*_(30)_ = −2.23, *p* = 0.033], and the number of errors did not differ between conditions [*T*_(30)_ = −1.05, *p* = 0.3, see Figures 1A,B]. Cortisol samples were analyzable for 27 out of 31 subjects. Increased cortisol levels under stress indicated a successful moderate stress induction [*T*_(26)_ = −2.7, *p* = 0.012]. Furthermore, negative affect ratings were higher in the stress condition [*T*_(30)_ = −5.69, *p* < 0.001], while positive affect ratings did not differ significantly [*T*_(30)_ = 1.5, *p* = 0.15, see Figures 1C,D].

### EEG

**Figure 2:**
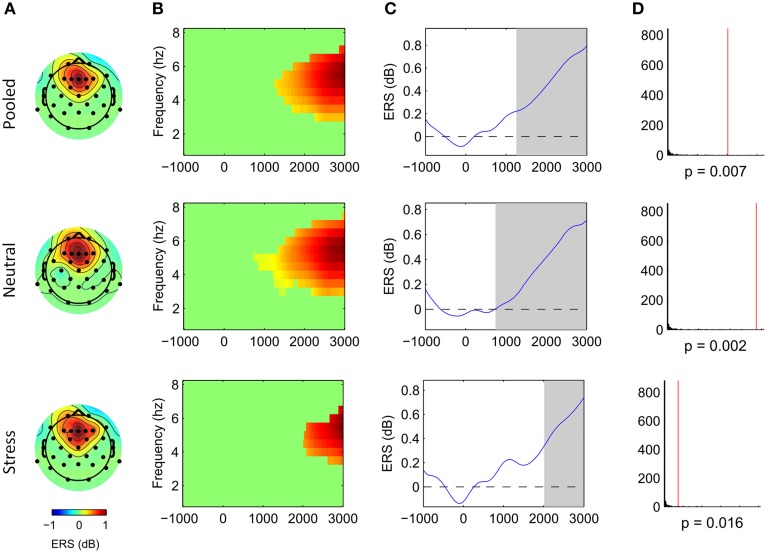
**Frontal midline theta increases during mental arithmetic in the stimulus-locked time window**. Cluster dimensions in the electrode-time-frequency space for pooled, neutral and stress conditions. **(A–C)** Show event-related synchronization (ERS) compared to a pre-stimulus baseline period (−1000 to 0 ms) in dB. **(A)** Shows the cluster dimensions in the electrode space. **(B)** Shows the cluster dimensions in the time-frequency space. **(C)** Shows the time course of the ERS. **(D)** Shows the distribution of random clusters in the cluster-based permutation test. The red line marks the position of the observed cluster.

**Figure 3:**
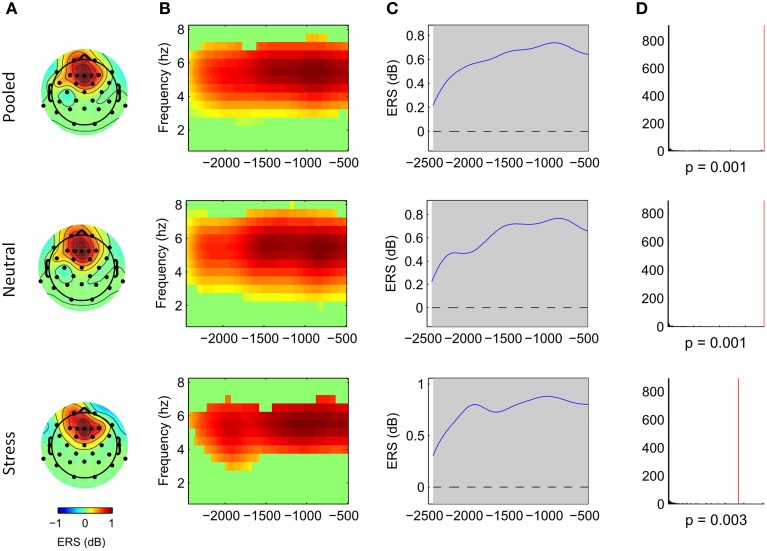
**Frontal midline theta increases during mental arithmetic in the response-locked time window**. Cluster dimensions in the electrode-time-frequency space for pooled, neutral and stress conditions. **(A–C)** Show event-related synchronization (ERS) compared to a pre-stimulus baseline period (−1000 to 0 ms) in dB. **(A)** Shows the cluster dimensions in the electrode space. **(B)** Shows the cluster dimensions in the time-frequency space. **(C)** Shows the time course of the ERS. **(D)** Shows the distribution of random clusters in the cluster-based permutation test. The red line marks the position of the observed cluster.

Cluster analysis revealed significant power increases from baseline in both, the stimulus-locked and the response-locked time window. In the neutral condition and stimulus-locked time window one significant cluster (*p* = 0.002, cluster statistic, Figure 2) was observed. Cluster dimensions ranged from 3 to 7.5 Hz in the frequency domain, and from 740 to 3000 ms in the time domain. In the neutral condition and response-locked window one significant cluster (*p* = 0.001, cluster statistic, Figure 3) that ranged from 2 to 8 Hz in the frequency domain, and from −2500 to −500 ms in the time domain, was observed. In the stress condition and stimulus-locked time window one cluster reached significance (*p* = 0.016, cluster statistic, Figure 2). It ranged from 3.5 to 6.5 Hz in the frequency domain, and from 2015 to 3000 ms in the time domain. In the stress condition and response-locked window the observed significant cluster (*p* = 0.003, cluster statistic, Figure 3) incorporated frequencies from 3 to 7 Hz, and time bins from −2500 to −500 ms. In the electrode space all clusters were located over a frontal midline region centered around Fz. All cluster statistics and dimensions are summarized in Table 1. Control time-frequency analyses without ERP removal showed an additional cluster in the theta frequency range. It ranged from 1.5 to 7 Hz in the frequency range, and from 0 to 615 ms in the time domain. It was located over the complete posterior half of the cortex, centered at bilateral parieto-occipital electrodes (see Supplementary Figure 1). Exploratory time-frequency analyses for higher frequencies (8–50 Hz) revealed strong decreases in the frequency range from 8 to 48 Hz, which was strongest in the alpha (8–13 Hz), and beta (15–25 Hz) band (see Supplementary Figure 2). The decrease started in the stimulus-locked time window at stimulus onset, and remained decreased until the end of the response-locked time window. It was located over the entire posterior half of the cortex, and strongest at occipital electrode sites.

**Table 1:** **Cluster statistics and dimensions**.

**Condition**	**Window**	**Clustersize (T-sum)**	***P*-value**	**Frequency range (Hz)**	**Time window (ms)**	**Location**
Pooled	Stimulus	10,118	0.007^**^	3–7	1255–3000	FM
Neutral	Stimulus	14,154	0.002^**^	3–7.5	740–3000	FM
Stress	Stimulus	3722	0.016^*^	3.5–6.5	2015–3000	FM
Neutral vs. Stress	Stimulus	707	0.022^*^	5.5–7.5	1670–2160	LF, FM
Pooled	Response	30,815	0.001^***^	2.5–7.5	−2500 to −500	FM
Neutral	Response	37,159	0.001^***^	2–8	−2500 to −500	FM
Stress	Response	18,543	0.003^**^	3–7	−2500 to −500	FM
Neutral vs. Stress	Response	–	–	–	–	–

*Clustersize values are based on the sum of all t-values in the observed clusters. P-values are determined by the number of random permutation clusters that showed a larger clustersizes than the observed clusters. Location abbreviations used: LF, left-frontal; FM, frontal-midline. Significant p-values are marked*:

* p < 0.05;

** p < 0.01;

*** *p < 0.001*.

**Figure 4:**
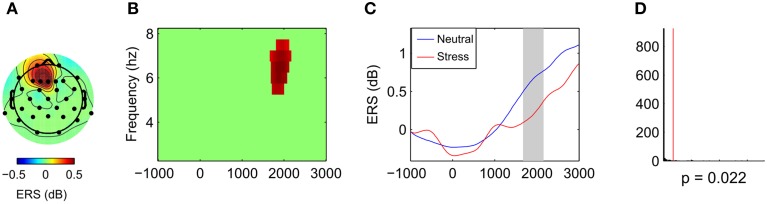
**Effects of stress on frontal midline theta during mental arithmetic (stimulus-locked time window)**. Cluster dimensions in the electrode-time-frequency space for neutral vs. stress condition. **(A–C)** Show differences in event-related synchronization (ERS) between the neutral and the stress condition in dB. **(A)** Shows the cluster dimensions in the electrode space. **(B)** Shows the cluster dimensions in the time-frequency space. **(C)** Shows the time course of the ERS. **(D)** Shows the distribution of random clusters in the cluster-based permutation test. The red line marks the position of the observed cluster.

Comparison of the neutral and the stress condition revealed one significant cluster that showed stronger power increases in the neutral condition (*p* = 0.022, cluster statistic, Figure 4). Cluster dimensions ranged from 5.5 to 7.5 Hz in the frequency domain, and from 1670 to 2160 ms in the time domain. In the electrode space the cluster was located over a frontal midline region (Fz) with a slight tendency toward the left hemisphere (F1 and F3).

**Figure 5:**
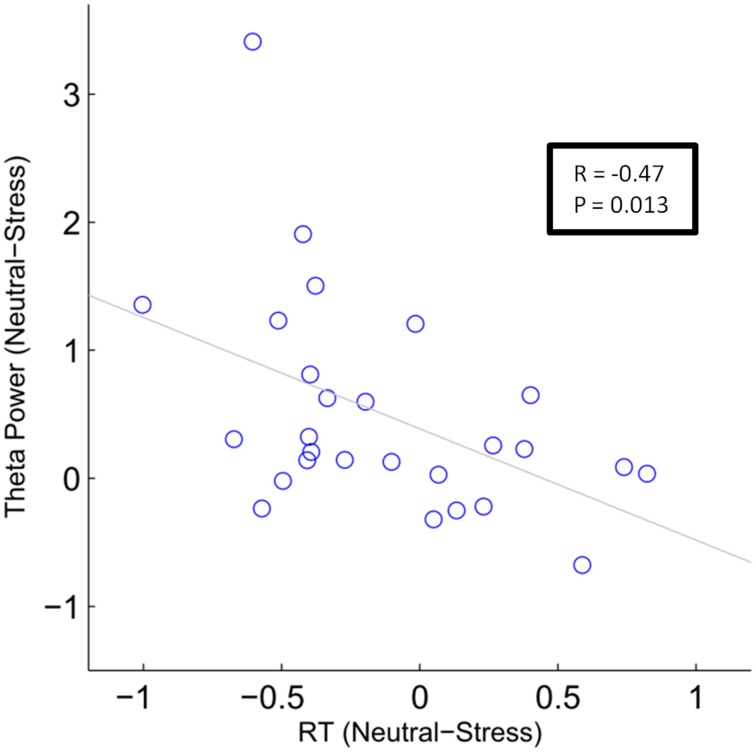
**Correlation analysis of stress effects during mental arithmetic**. Differences in frontal midline theta activity as a function of differences in reactions times. The blue circles depict single subject values (*N* = 27). The gray line depicts the least squares regression line.

To test whether this difference in FMT activity was related to differences in behavioral performance, we conducted a between-subject correlation analysis (Pearson's correlation coefficient). For each subject the mean difference between conditions (neutral—stress) was calculated for FMT activity and reaction times. For FMT activity, the mean cluster value was calculated. Results showed that decreased FMT activity under stress was related to slower reaction times under stress. Applied to all 31 subjects the correlation was marginally significant (*r* = −0.31, *p* = 0.088). The correlation reached significance (*r* = −0.47, *p* = 0.013, Figure 5) if four subjects that did not show FMT increases (compared to baseline) were excluded.

## Discussion

In this study, we investigated FMT oscillations during a mental arithmetic task that was carried out in a stressful and a neutral control condition. We found late-onset, sustained FMT increases during mental arithmetic compared to a pre-stimulus baseline period. In the stress condition, the onset of the FMT response was delayed. This difference was quantified by stronger FMT increases in the neutral condition in an early time window.

The increase in the theta band (3–8 Hz) we observed during mental arithmetic was characterized by a late onset (>700 ms), and a long duration (>2000 ms). It was not phase-locked to the presented stimuli (induced oscillations), and clearly located over the frontal midline region (centered around Fz). A large number of studies have reported such frontal midline theta (FMT) increases during different cognitive tasks. Several studies have used source localization techniques to identify possible generators of FMT (Gevins et al., 1997; Asada et al., 1999; Onton et al., 2005). These studies suggest that FMT could be generated in the anterior cingulate cortex (ACC), and medial prefrontal cortex (mPFC). Despite the differences between cognitive tasks that elicit FMT, it has been proposed that sustained, internally-directed cognition that is independent from external stimuli or responses could pose a potential common denominator (Hsieh and Ranganath, 2014). During the mental arithmetic task we applied, this could represent the process of maintaining and manipulating single digits while adding up two 3-digit numbers. The late onset of the FMT response we report is in favor of this interpretation. It can be expected that encoding of the stimuli is completed prior to the onset of the observed FMT increase.

The maintenance and manipulation of digits during mental arithmetic is closely related to processes involved in WM (Imbo et al., 2007). During WM, it has been shown that FMT increases with workload (Gevins et al., 1997; Jensen and Tesche, 2002). This is in line with the continuous increase of FMT we observed. During the process of adding up two 3-digit numbers, an increasing number of digits has to be kept in mind, which leads to increasing workload across the time course of the task. Although the relationship between FMT and WM processes is well-established, a functional interpretation is still lacking. For example, it remains an open question whether FMT plays a particular role in the maintenance of information, such as maintaining temporal sequence information in multi-item working memory paradigms (Hsieh et al., 2011), or whether it rather provides a general framework in which task-irrelevant information is inhibited to optimize performance (Scheeringa et al., 2009).

We found a decreased FMT response under stress. This finding is consistent with the results of a recently published study that observed stress-related FMT decreases during a working memory task (Gaertner et al., 2014). In contrast to this study, a detailed time course analysis of the FMT response was conducted here. The effect of stress on FMT was present in a time window ranging from 1670 to 2160 ms. It can be assumed that FMT in this time window is related to internally-directed mental operations, independent from stimulus- or response processing. Differences between conditions in FMT activity were only observed in the stimulus-locked time window, and not in the response-locked window. Furthermore, FMT started to increase much earlier in neutral condition (740 ms after stimulus presentation) than in the stress condition (2015 ms after stimulus presentation), and slowed reaction times under stress were associated with stress-related FMT decreases. A possible explanation could be that under stress subjects experienced difficulties to reach a state of focused attention and suppress task-irrelevant information. This assumption receives support from a study by Qin et al. (2009) who found impaired DMN suppression in a WM task that was carried out under stress. Given the inverse relationship between DMN activity and FMT, this finding supports the view that FMT oscillation during demanding cognitive tasks might provide a mechanism by which fragile temporally stored information is protected from internal and external distractions.

Additional oscillatory signatures that were observed during mental arithmetic, such as the transient, stimulus-evoked increase in the theta frequency range, and decreases in higher frequency ranges, did not show differences between the neutral and the stress condition. A possible explanation could be that these EEG signatures are related to basic visual processing. The early onset of these responses, and their location over posterior electrode sites support this assumption. It is well-established that the PFC is the brain region most vulnerable to the detrimental effects of stress (Arnsten, 2009). Since there is some evidence suggesting that FMT is generated in the PFC, our results support this line of evidence.

Our study had some limitations that have to be addressed in upcoming studies. The number of electrodes was rather small (31 electrodes). To further investigate precise topographic differences between the different types of FMT, high-density electrode arrays that allow for a more precise localization (and source localization) should be applied. Furthermore, we only recruited male subjects to control for effects of the hormonal cycle on the stress induction. Upcoming studies should investigate whether the observed effects can be generalized to both genders.

In conclusion, our results show late-onset, sustained FMT increases during mental arithmetic, and suggest that these increases are related to stimulus-independent mental processes. Externally induced stress reduced FMT during mental arithmetic, possibly indicating difficulties to focus attention and to suppress task-irrelevant information. As such, FMT poses a potential marker for intact PFC function that could prove useful in the treatment of stress-related diseases.

### Conflict of interest statement

The authors declare that the research was conducted in the absence of any commercial or financial relationships that could be construed as a potential conflict of interest.
