# Supplementary figures and images for: Frontal midline theta oscillations during mental arithmetic: effects of stress
Source: Front Behav Neurosci. 2015 Apr 20;9:96. doi: 10.3389/fnbeh.2015.00096 (PMC4403551; doi:10.3389/fnbeh.2015.00096)

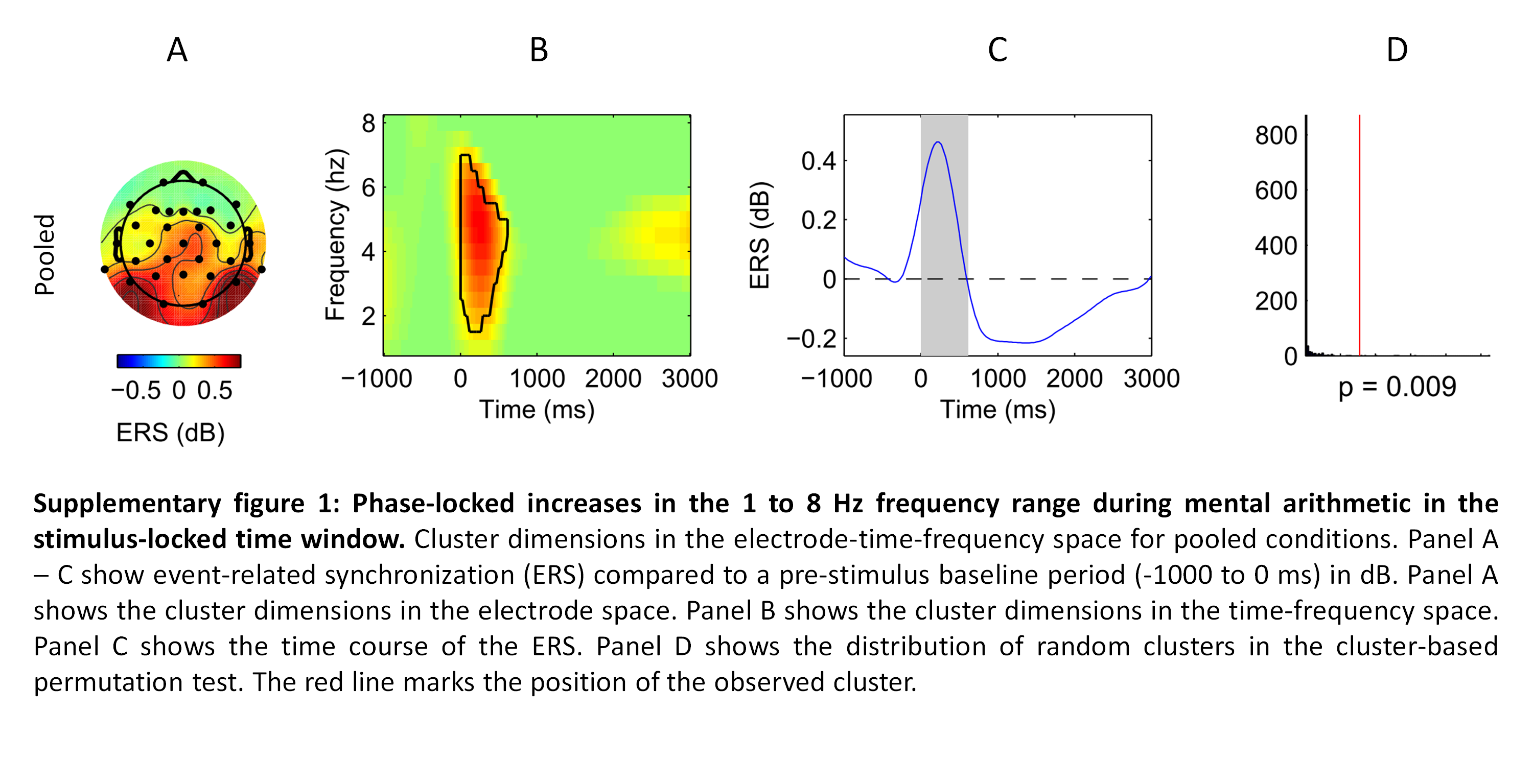

Supplement: Supplementary file 1 [file Image1.TIF]

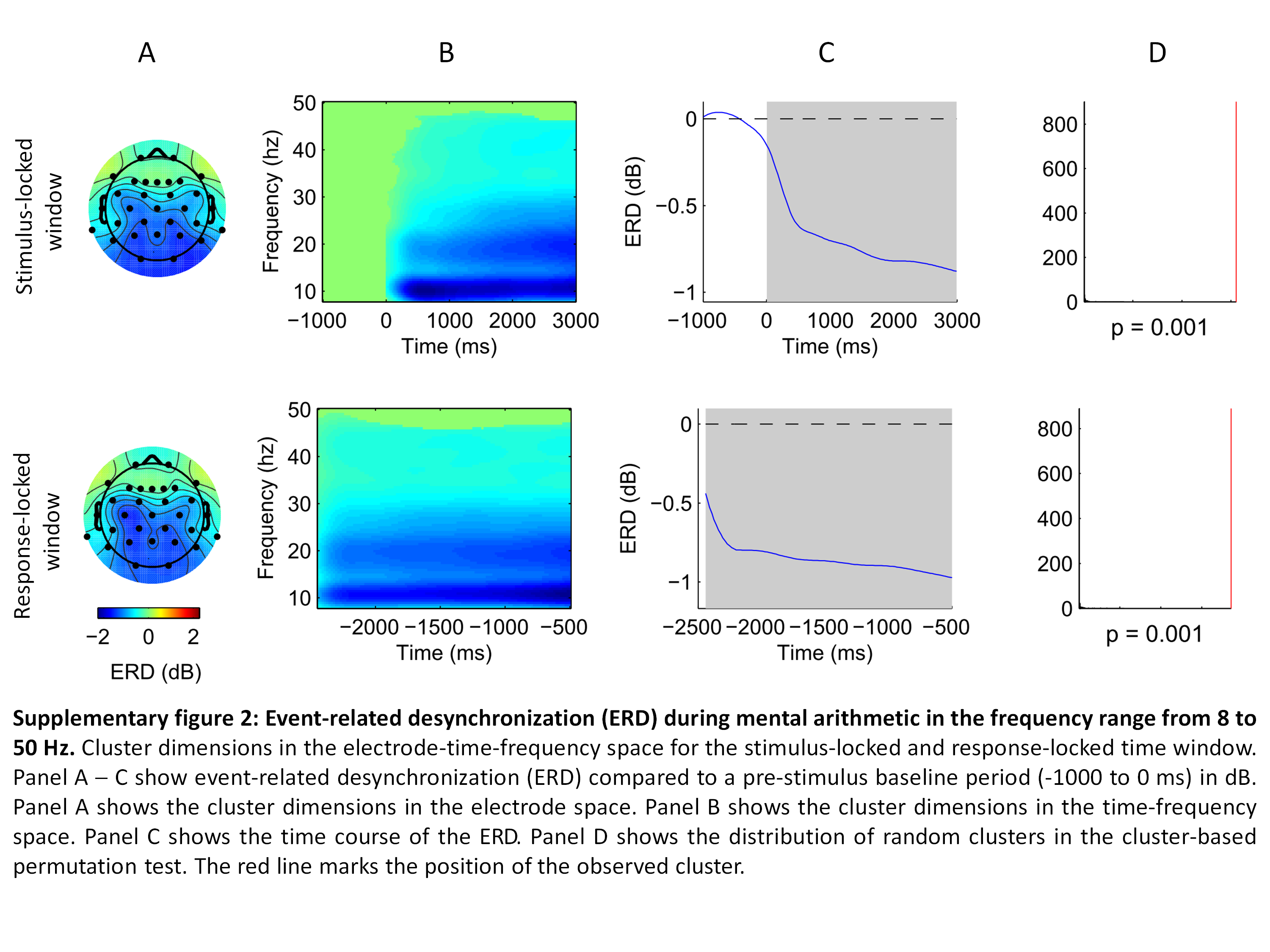

Supplement: Supplementary file 2 [file Image2.TIF]
